# Genetic Polymorphisms of Glutathione S-Transferase Genes GSTM1, GSTT1 and Risk of Hepatocellular Carcinoma

**DOI:** 10.1371/journal.pone.0048924

**Published:** 2012-11-20

**Authors:** Kang Song, Jiayong Yi, Xizhong Shen, Yu Cai

**Affiliations:** 1 Liver Cancer Institute, Zhongshan Hospital, Fudan University, Shanghai, People’s Republic of China; 2 Department of Orthopedics, Zhongshan Hospital, Fudan University, Shanghai, People’s Republic of China; 3 Department of Gastroenterology, Zhongshan Hospital, Fudan University, Shanghai, People’s Republic of China; Sanjay Gandhi Medical Institute, India

## Abstract

**Background:**

A number of case-control studies were conducted to investigate the association of glutathione S-transferase (GST) genetic polymorphisms and hepatocellular carcinoma (HCC) risk. However, these studies have yielded contradictory results. We therefore performed a meta-analysis to derive a more precise estimation of the association between polymorphisms on GSTM1, GSTT1 and HCC.

**Methodology/Prinicpal Findings:**

PubMed, EMBASE, ISI web of science and the CNKI databases were systematically searched to identify relevant studies. Data were abstracted independently by two reviewers. Odds ratios (ORs) and 95% confidence intervals (95% CIs) were used to assess the strength of association. Potential sources of heterogeneity were also assessed by subgroup analysis and meta-regression. Funnel plots and Egger’s linear regression were used to test publication bias among the articles. A total of 34 studies including 4,463 cases and 6,857 controls were included in this meta-analysis. In a combined analysis, significantly increased HCC risks were found for null genotype of GSTM1 (OR = 1.29, 95% CI: 1.06–1.58; P = 0.01) and GSTT1 (OR = 1.43, 95% CI: 1.22–1.68; P<10^−5^). Potential sources of heterogeneity were explored by subgroup analysis and meta-regression. Significant results were found in East Asians and Indians when stratified by ethnicity; whereas no significant associations were found among Caucasians and African populations. By pooling data from 12 studies that considered combinations of GSTT1 and GSTM1 null genotypes, a statistically significant increased risk for HCC (OR = 1.88, 95% CI: 1.41–2.50; P<10^−4^) was detected for individuals with combined deletion mutations in both genes compared with positive genotypes.

**Conclusions/Significance:**

This meta-analysis suggests that the GSTM1 and GSTT1 null genotype may slightly increase the risk of HCC and that interaction between unfavourable GSTs genotypes may exist.

## Introduction

Hepatocellular carcinoma (HCC) is the fifth most common cancer and the third cause of cancer-related death worldwide [1]. The etiologic importance of chronic infection with hepatitis B virus (HBV) and hepatitis C virus (HCV) in HCC has been well established [2], [3]. Not only is HCC an inevitable consequence of chronic HBV or HCV infection, but also other HCC risk factors, such as tobacco smoking, alcohol drinking and aflatoxin exposure, are related to susceptibility to HCC [4], [5]. However, only a minority of patients at risk develops HCC and it is likely that other risk factors such as environmental carcinogenic compounds may contribute to HCC development. This has maintained interest in other biochemical and genetic factors that might contribute to the underlying pathophysiology of HCC.

The glutathione S-transferases (GSTs) are a gene superfamily of phase II metabolic enzymes that detoxify free radicals, particularly in tobacco smoke, products of oxidative stress, and carcinogens such as benzopyrene and other polycyclic aromatic hydrocarbons. The most potent mutagenic and carcinogenic of the aflatoxins is aflatoxins B1 (AFB1) which is mainly metabolized by cytochrome P450 3A4 into the genotoxic metabolite AFB1–8,9-exo-epoxide. This metabolite can bind to DNA, causing G-to-T transversions [6] that may ultimately lead to cancer. Detoxification prevents formation of DNA adducts; the metabolite may be conjugated to glutathione by GSTs or may be hydrolyzed. In addition to their role in phase II detoxification, GSTs also play an important role in modulating the induction of other enzymes and proteins for cellular functions, such as DNA repair [7]. GSTM1 and GSTT1 are the most extensively studied genes in the GST gene superfamily. Polymorphic deletion variants in the GSTM1 and GSTT1 genes produce either a functional enzyme (non-deletion alleles or heterozygous deletion, GSTM1-1 and GSTT1-1) or result in the complete absence of the enzyme (homozygous deletion alleles, GSTM1-null and GSTT1-null) [8]. Therefore, these enzymes may be related to the risk for HCC.

Over the past few years, considerable efforts have been devoted to exploring the relationships between the GSTT1 and GSTM1 null polymorphisms and HCC risk among various populations. However, existing studies have yielded inconsistent results. These disparate findings may be due partly to insufficient power, false-positive results and publication biases. The interpretation of these studies has been further complicated by the use of different control source. In addition, with the increased studies in recent years among Asian, Caucasian, and other populations, there is a need to reconcile these data. We therefore performed a meta-analysis of the published studies to clarify this inconsistency and to establish a comprehensive picture of the relationship between GSTM1, GSTT1 and HCC.

## Materials and Methods

### Literature Search Strategy and Selection Criteria

Genetic association studies published before the end of June 2012 on HCC and polymorphisms in the *GST* gene were identified through a search of PubMed, Web of Science, EMBASE and CNKI (Chinese National Knowledge Infrastructure). Search term were keywords relating to the relevant gene (e.g. ‘glutathione S-transferase’, ‘*GST*’, *GSTM1*’, *GSTT1*’) in combination with words related to liver cancer (e.g. ‘Hepatocellular carcinoma’, ‘Liver neoplasm’, ‘Liver cancer’) and polymorphism or variation. Furthermore, reference lists of main reports and review articles were also reviewed by a manual search to identify additional relevant publications.

The included studies have to meet the following criteria: (1) original papers containing independent data, (2) identification of HCC patients was confirmed histologically or pathologically, (3) genotype distribution information or odds ratio (OR) with its 95% confidence interval (CI) and *P*-value, (4) case–control or cohort studies. The major reasons for exclusion of studies were (1) overlapping data and (2) case-only studies, family based studies, and review articles.

### Eligible Studies and Data Extraction

Data extraction was performed independently by two reviewers and differences were resolved by further discussion among all authors. For each included study, the following information was extracted from each report according to a fixed protocol: first author, publication year, definition and numbers of cases and controls, diagnostic criterion, frequency of genotypes, age, sex, cigarette smoking status, alcohol drinking, ethnicity and genotyping method.

### Statistical Methods

For the GSTM1 and GSTT1 gene, we estimated the risks of the null genotype on HCC, compared with the non-null genotypes in the recessive model. The strength of the association between the GSTM1 and GSTT1 gene and HCC risk was measured by odds ratios (ORs) with 95% confidence intervals (CIs).

Heterogeneity across individual studies was calculated using the Cochran chi-square Q test followed by subsidiary analysis or by random-effects regression models with restricted maximum likelihood estimation [9], [10]. Random-effects and fixed-effect summary measures were calculated as inverse variance-weighted average of the log OR. The results of random-effects summary were reported in the text because it takes into account the variation between studies. In addition, sources of heterogeneity were investigated by stratified meta-analyses based on ethnicity, source of controls (population or hospital based) and sample size (No. cases ≥200 or <200). Ethnic group was defined as East Asians, Caucasians (i.e. people of European origin), Indians and Africans. 95% CIs were constructed using Woolf’s method [11]. The significance of the overall OR was determined by the Z-test. Ethnicity, sample size, control source, sex distribution among cases and controls were analyzed as covariates in meta-regression. Funnel plots and Egger’s linear regression test were used to assess evidence for potential publication bias [12]. In order to assess the stability of the result, sensitivity analyses were performed, each study in turn was removed from the total, and the remaining were reanalyzed. All statistical analyses were carried out with the Stata software version 10.0 (Stata Corporation, College Station, TX, USA). The type I error rate was set at 0.05 for two-sided analysis.

## Results

### Characteristics of Studies

The combined search yielded 287 references. 254 articles were excluded because they clearly did not meet the criteria or overlapping references (Figure S1). Finally, a total of 34 studies were retrieved based on the search criteria for HCC susceptibility related to the GST polymorphisms [13]–[46]. Study quality was assessed according to the score scale for randomized controlled association study proposed by Clark et al [47]. The main study characteristics were summarized in Table 1. There are 33 studies with 4412 cases and 6804 controls concerning GSTM1 and 28 studies with 3892 HCC cases and 6117 controls concerning GSTT1. Of the cases, 74% were East Asians, 10% were Caucasians, 9% were Indians and 7% were African.

**Table 1:** Characteristics of the studies included in the meta-analysis.

Study	Year	Ethnicity	No. of cases	No. of controls	Source ofcontrol	Sex incases(male%)	Sex in controls (male%)	Genotypingmethod	Quality score
Dong [13]	1997	Chinese	110	112	Population	83.0	84.7	PCR	7
Yu [14]	1999	Chinese	84	375	Population	100	100	PCR	5
Wu [15]	2000	Chinese	54	136	Population	85.2	NA	PCR	8
Huang [16]	2000	Chinese	83	107	Population	NA	NA	PCR	7
Zhu [17]	2001	Chinese	52	100	Population	NA	NA	MD-PCR	8
Sun [18]	2001	Chinese	69	128	Population	83.5	81.9	PCR	6
Tiemersma [19]	2001	Sudanese	112	194	Population	76.8	75.3	Multiplex PCR	7
Liu [20]	2002	Chinese	84	144	Population	NA	NA	Multiplex PCR	8
Chen [21]	2002	Chinese	101	35	Hospital	91.3	48.6	PCR	5
Munaka [22]	2003	Japanese	77	138	Hospital	78.2	68.1	PCR	10
Wu [23]	2003	Chinese	62	58	Population	NA	NA	PCR	6
Liu [24]	2003	Chinese	51	53	Hospital	86.3	77.3	PCR	8
Yu [25]	2003	Chinese	577	389	Population	86.0	86.0	PCR	9
McGlynn [26]	2003	Chinese	231	256	Population	80.9	73.8	PCR	9
Li [27]	2004	Chinese	207	207	Population	81.6	81.6	PCR	8
Deng [28]	2005	Chinese	181	360	Hospital	80.1	NA	PCR	8
Covolo [29]	2005	Italian	200	400	Hospital	NA	NA	Multiplex PCR	9
Zhu [30]	2005	Chinese	91	130	Population	86.8	87.3	Multiplex PCR	7
Ma [31]	2005	Chinese	62	73	Population	69.3	69.9	PCR	7
Kirk [32]	2005	Gambian	194	352	Hospital	80.1	71.6	Multiplex PCR	8
Long [33]	2005	Chinese	140	536	Hospital	79.3	71.6	Multiplex PCR	9
Zhang [34]	2005	Chinese	60	73	Population	76.7	76.7	PCR	5
Guo [35]	2005	Chinese	95	103	Population	74.7	67.0	Multiplex PCR	8
Ladero [36]	2006	Spanish	184	329	Population	81.5	60.2	PCR	9
Long [37]	2006	Chinese	257	649	Hospital	80.9	75.5	PCR	8
Borentain [38]	2007	French	56	79	Population	87.5	67.5	PCR	10
Kiran [39]	2008	Indian	63	169	Hospital	90.5	72.2	Duplex PCR	7
He [40]	2008	Chinese	105	151	Population	91.4	90.7	PCR	6
Moneim [41]	2008	Egyptian	60	50	Hospital	NA	NA	PCR	8
Imaizumi [42]	2009	Japanese	209	256	Hospital	67.5	65.2	PCR	9
Yang [43]	2009	Chinese	100	60	Hospital	84.0	78.3	Multiplex PCR	5
Asim [44]	2010	Indian	254	525	Hospital	79.5	70.7	PCR	7
Xiao [45]	2011	Chinese	130	75	Population	NA	NA	Multiplex PCR	8
Sarma [46]	2012	Indian	68	55	Hospital	83.8	58.2	PCR	7

NA: not available.

### Association of GSTM1null Polymorphism with HCC

**Figure 1:**
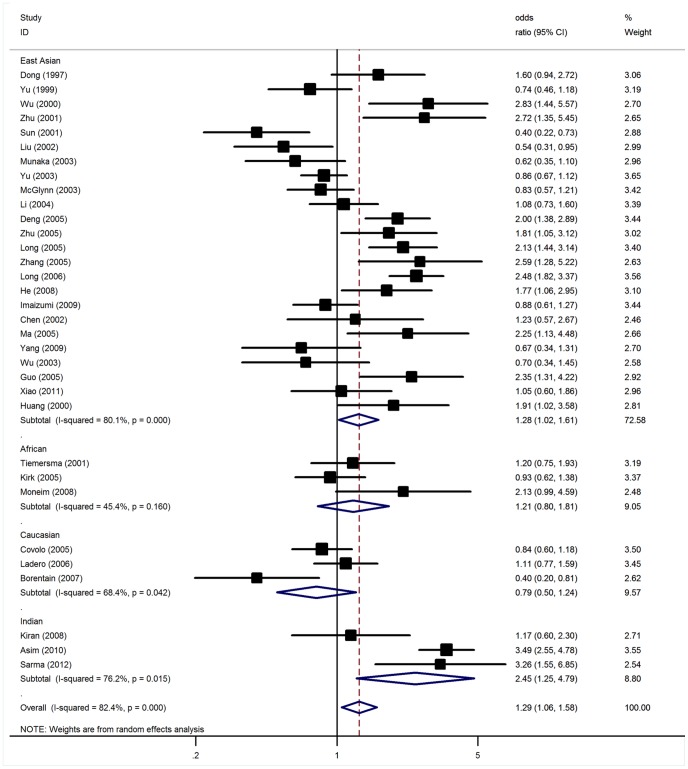
Meta-analysis of GSTM1 null genotype associated with HCC.

Overall, there was evidence of an association between the increased risk of HCC and the null genotype of GSTM1 when all eligible studies were pooled into the meta-analysis. Using random effect model, the summary OR of GSTM1 null genotype was 1.29 (95% CI: 1.06–1.58, *P* = 0.01, Figure 1) with statistically significant between-study heterogeneity (*P*<10^−5^). This analysis is based on pooling of data from a number of different ethnic populations. When stratifying for ethnicity, significant associations were found for East Asians and Indians with OR of 1.28 (95% CI: 1.02–1.61, *P* = 0.03) and of 2.45 (95% CI: 1.25–4.79, *P* = 0.009), respectively. Subsidiary analyses of control source yielded an OR for hospital-based controls of 1.44 (95% CI: 1.02–2.02) and for population-based controls of 1.19 (95% CI: 0.95–1.50). When studies were stratified for sample size, significant risk was found in small studies with OR of 1.30 (95% CI: 1.04–1.62, *P* = 0.02). However, no significant association was detected among seven large studies (Table 2). In meta-regression analysis, ethnicity (*P* = 0.63), sample size (*P* = 0.29), source of controls (*P* = 0.45), sex distribution in cases (*P* = 0.27) and controls (*P* = 0.47) did not significantly explained such heterogeneity.

**Table 2:** Main results of pooled odds ratios (ORs) with confidence interval (CI) in the meta-analysis.

Sub-group analysis	GSTM1				GSTT1			
	No. ofcase/control	OR (95%CI)	P(Z)	P(Q)	No. ofcase/control	OR (95%CI)	P(Z)	P(Q)
Overall	4412/6804	1.29 (1.06–1.58)	0.01	<10^−5^	3892/6117	1.43 (1.22–1.68)	<10^−5^	<10^−5^
Ethnicity								
East Asian	3221/4651	1.28 (1.02–1.61)	0.03	<10^−4^	2869/4237	1.40 (1.18–1.66)	<10^−4^	<10^−5^
Caucasian	440/808	0.79 (0.50–1.24)	0.31	0.04	384/729	1.09 (0.71–1.68)	0.68	0.16
Indian	385/749	2.45 (1.25–4.79)	0.009	0.02	385/749	1.96 (0.96–3.98)	0.06	0.01
African	366/596	1.21 (0.80–1.81)	0.37	0.16	254/402	1.54 (0.72–3.31)	0.27	0.08
Sample size								
<200	2477/4122	1.30 (1.04–1.62)	0.02	<10^−4^	2166/3691	1.48 (1.24–1.75)	<10^−5^	0.002
≥200	1935/2682	1.26 (0.80–2.00)	0.32	<10^−4^	1726/2426	1.30 (0.89–1.91)	0.17	<10^−4^
Control source								
Hospital	2527/2541	1.44 (1.02–2.02)	0.04	<10^−4^	1645/3347	1.66 (1.30–2.12)	<10^−5^	<10^−4^
Poulation	2508/3219	1.19 (0.95–1.50)	0.13	<10^−4^	2247/2770	1.28 (1.05–1.55)	0.01	0.002

### Association of GSTT1null Polymorphism with HCC

**Figure 2:**
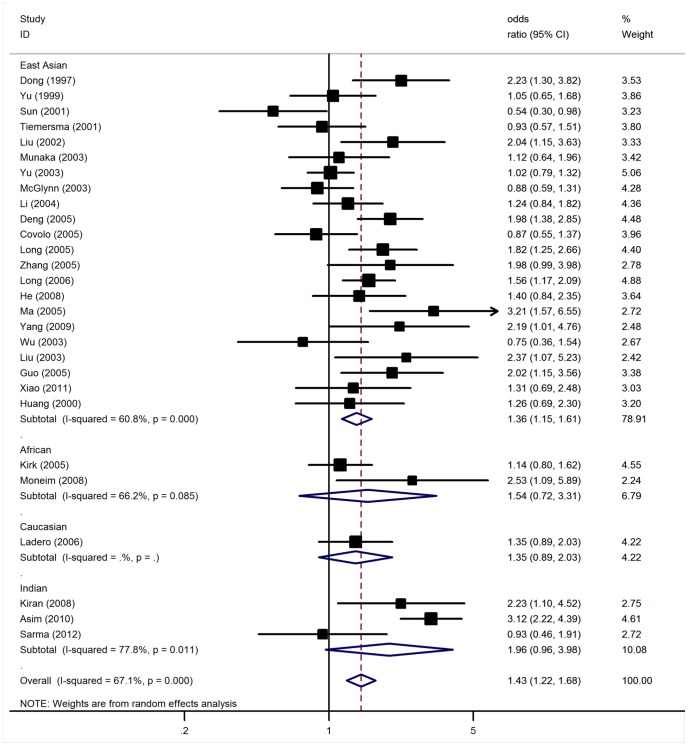
Meta-analysis of GSTT1 null genotype associated with HCC.

Significant heterogeneity was present among the 28 studies (*P*<10^−5^). In a meta-regression analysis, ethnicity (*P* = 0.53), sample size (*P* = 0.32), source of controls (*P* = 0.14), and gender distribution among cases (*P* = 0.30) and controls (*P* = 0.38) explained little heterogeneity. Overall, significantly increased HCC risk was found for GSTT1 null genotype (OR = 1.43, 95% CI: 1.22–1.68, *P*<10^−5^; Figure 2).

In the subgroup analyses by ethnicity, significant risks were found among East Asians with OR of 1.40 (95% CI: 1.18–1.66, *P*<10^−4^). However, no such associations were detected among Caucasians, African and Indian populations (Table 2). Subsidiary analyses of sample size yielded an OR for small studies of 1.48 (95% CI: 1.24–1.75, *P*<10^−5^), while no significant results were found for large studies. By considering control source subgroups, the OR was 1.28 (95% CI: 1.05–1.55, *P* = 0.01) in population-based controls, compared to 1.66 (95% CI: 1.30–2.12, P<10^−5^) in hospital controls.

### Gene–gene Interaction

**Figure 3:**
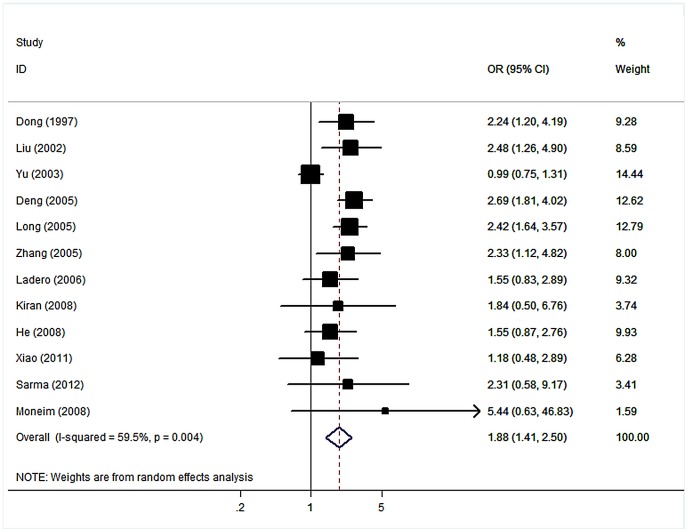
Forest plot for association of dual null genotype of GSTM1/GSTT1 and HCC risk.

The effect of each genotype of GSTs was independently assessed. Significant association was established between the null genotype of GSTM1, GSTT1 and HCC. The data on both null genotype of GSTs among cases and controls were available in 12 studies which included 1762 cases and 2443 controls. The interaction between GSTM1 null and GSTT1 null, for which an OR of 1.88 (95% CI: 1.41–2.50, *P*<10^−4^; figure3) for HCC appeared in compared with individuals with the positive genotypes.

### Sensitivity Analyses and Publication Bias

A single study involved in the meta-analysis was deleted each time to reflect the influence of the individual data-set to the pooled ORs, and the corresponding pooled ORs were not qualitatively altered. Begg’s funnel plot and Egger’s test were performed to access the publication bias of the literatures. The shape of the funnel plots was symmetrical for these polymorphisms (Figure S2 and S3). The statistical results still did not show publication bias in these studies for GSTM1 (Egger test, *P* = 0.90) and GSTT1 (Egger test, *P* = 0.55).

## Discussion

Large sample and unbiased epidemiological studies of predisposition genes polymorphisms could provide insight into the in vivo relationship between candidate genes and diseases. This is the most comprehensive meta-analysis that examined the GSTM1, GSTT1 null polymorphism and the relationship to susceptibility for hepatocellular carcinoma. Its strength was based on the accumulation of published data giving greater information to detect significant differences. In total, the meta-analysis involved 34 studies for hepatocellular carcinoma, which provided 4463 cases and 6857 controls.

Our results demonstrated that the GSTM1 and GSTT1 null polymorphism is a risk factor for developing hepatocellular carcinoma. However, this association became non-significant when the meta-analysis was restricted to larger studies, suggesting potential small studies effects. In the stratified analysis by ethnicity, significant associations were found in East Asians for the two polymorphisms. Significant result was also found among Indians for GSTT1 null polymorphism. However, no significant associations were detected among Caucasian and African populations for the two polymorphisms. There are several possible reasons for such differences. Firstly, the frequencies of the risk-association homozygous null genotype vary between different races. For example, the homozygous null genotype distributions of the GSTT1 polymorphism varies between East Asian, Indian and African populations, with a prevalence of ∼52%, ∼29%, and ∼31%, respectively [19], [22], [39]. Therefore, additional studies are warranted to further validate ethnic difference in the effect of these polymorphisms on HCC risk. Secondly, such different results could also be explained by study design or sample size. Besides, other confounding factors, such as age, sex, life style should also be considered. Thus, further prospective researches are needed to examine the effect of this variation on HCC to make a comprehensive and true conclusion [18]. Other confounding factors, such as age, life style (e.g. smoking, alcohol consumption) should also be considered [22], [27], [36]. Nevertheless, owing to the limited number of relevant studies among Caucasians and Africans included in this meta-analysis, the observed ethnic difference in this meta-analysis is also likely to be caused by chance because studies with small sample sizes may have insufficient statistical power to detect a slight effect or may have generated a fluctuated risk estimate. Thus, further studies including a wider spectrum of subjects to investigate the role of this variant in these populations will be needed.

Our results indicated that significantly increased HCC risk in GSTM1 null genotype was found among the hospital-based studies but not in population-based studies. Possible reason could be that the hospital-based studies have some biases because such controls may just represent a sample of ill-defined reference population, and may not be representative of the general population very well, particularly when the genotypes under investigation were associated with the disease conditions that the hospital-based controls may have. Therefore, using a proper and representative population-based control subjects is very important to reduce biases in such genetic association studies.

If genetic susceptibility to HCC is, in part, mediated through metabolic gene polymorphisms, it is possible that the combinations of certain genotypes may be more discriminating as risk factors for HCC than a single locus genotype. Among the 34 studies included in the present meta-analysis, 12 studies investigated the interaction between GSTM1 and GSTT1 polymorphism. By pooling the collected data on GSTM1 and GSTT1 genotypes, a statistically significant 1.88-fold increased risk for HCC appeared for individuals with combined deletion mutations in GSTT1 and GSTM1 genes in comparison with individuals with the positive genotypes. This result suggests that in the presence of both of the two risk factors, an important number of HCC cases would occur.

It is well accepted that the strength of an association is not an inherent biologic property with small associations potentially reflecting important causal relations. It may take relatively few common genetic variants, each conveying only small to modest excess risk, to account for a sizable portion of the population attributable fraction for common diseases (e.g., 10–18 genes, each with a 20–30 percent prevalence and conveying an odds ratio of only 1.2–1.5 to explain between 30 and 50 percent of the population attributable fraction [48]. Recently, several other drug metabolizing genes were found to be associated with HCC susceptibility. N-acetyltransferase 2 and CYP1A1 genotype were found to be associated with increased risk of HCC among smokers [49], [50]. Liu et al. reported that CYP2E1 PstI/RsaI polymorphism was significantly associated with increased HCC susceptibility among alcohol drinkers [51]. Therefore, these gene/polymorphisms which works in close network with each other should be address in future studies.

A number of factors predict HCC, however, detailed pathogenesis mechanisms of HCC remain a matter of speculation. Members of the GST family are important candidates for involvement in susceptibility to commonly occurring forms of cancer, because they may regulate an individual’s ability to metabolize environmental carcinogens [52]. Normal or increased GST enzyme activity or levels may protect susceptible tissues from somatic mutations in DNA by facilitating the conjugation and subsequent elimination of electrophilic carcinogens [53]. Absent or deficient GST enzyme activity may result in poorer elimination of electrophilic carcinogens, particularly in the presence of very active electrophilic activation by phase I enzymes. If an individual’s inherited genotype at a GST locus does not permit the efficient metabolism of compounds involved in carcinogens, then that individual may be at increased cancer risk. GSTM1 and GSTT1 are two important members of GSTs supergene family, which plays an important role in the second stage of biotransformation by conjugating extraneous chemicals with glutathione [54]. Previous some studies have showed that the deficient types of GSTM1 and GSTT1, namely GSTM1-null and GSTT1-null genotypes are completely lack of respective enzyme activity and cannot metabolize transfer AFB1-epoxide, the most important metabolic carcinogen of AFB1, to un-toxic metabolite which is highly soluble and can be excreted out of body, which may be associated with the increased risk of HCC [18], [32].

Compared with the previous meta-analysis [55]–[57], the present study is much larger. In addition, we assessed not only the effect of each genotype of GSTs and HCC independently but also the interaction effect between GSTM1 and GSTT1. Furthermore, we also performed meta-regression to evaluate the effect of several preselected factors on the observed variability among studies evaluating GSTM1 or GSTT1 null genotype. Our results suggest an overestimation of the true genetic association by small studies, consistent with the phenomenon known as “winner’s curse” [58], [59].

In interpreting the results, some limitations of this meta-analysis should be addressed. Firstly, heterogeneity is a potential problem when interpreting all the results of meta-analysis. Although we minimized the likelihood by performing a careful search for published studies, using the explicit criteria for study inclusion, the significant between-study heterogeneity still existed in most of comparison. The presence of heterogeneity can result from differences in the age distribution, selection of controls, prevalence lifestyle factors and so on. Secondly, the subgroup meta-analyses considering interactions between GSTM1, GSTT1 null genotype and ethnic difference, as well as between GSTT1 null and GSTM1 null genotypes, were performed on the basis of a fraction of all the possible data to be pooled, so selection bias may have occurred and our results may be over inflated. In this context, more reliable results can be expected if individual data are available for a pooled analysis. Finally, lacking the original data of the reviewed studies limited our further evaluation of potential interactions because the interactions between gene-environment, such aflatoxin B1 exposure, HBV or HCV infection, smoking and alcohol abuse.

Despite these limitations, this meta-analysis suggests that the null genotypes of GSTM1 and GSTT1 may increase the risk of HCC, particularly in East Asian population. In addition, the combination of unfavourable genotypes of GSTM1 and GSTT1 may result in an additional risk of HCC. For future association studies, strict selection of patients and controls, larger studies of different ethnic populations will be required. Moreover, gene–gene and gene–environment interactions should also be considered in future studies.

## Supporting Information

Figure S1 Flow diagram of the study selection process.
(TIF)

Figure S2 Begg’s funnel plot for publication bias in selection of studies on GSTM1 polymorphism.
(TIF)

Figure S3 Begg’s funnel plot for publication bias in selection of studies on GSTT1 polymorphism.
(TIF)

Checklist S1 (DOC)
