# Supplementary figures and images for: Genetic Polymorphisms of Glutathione S-Transferase Genes GSTM1, GSTT1 and Risk of Hepatocellular Carcinoma
Source: PLoS One. 2012 Nov 20;7(11):e48924. doi: 10.1371/journal.pone.0048924 (PMC3502401; doi:10.1371/journal.pone.0048924)

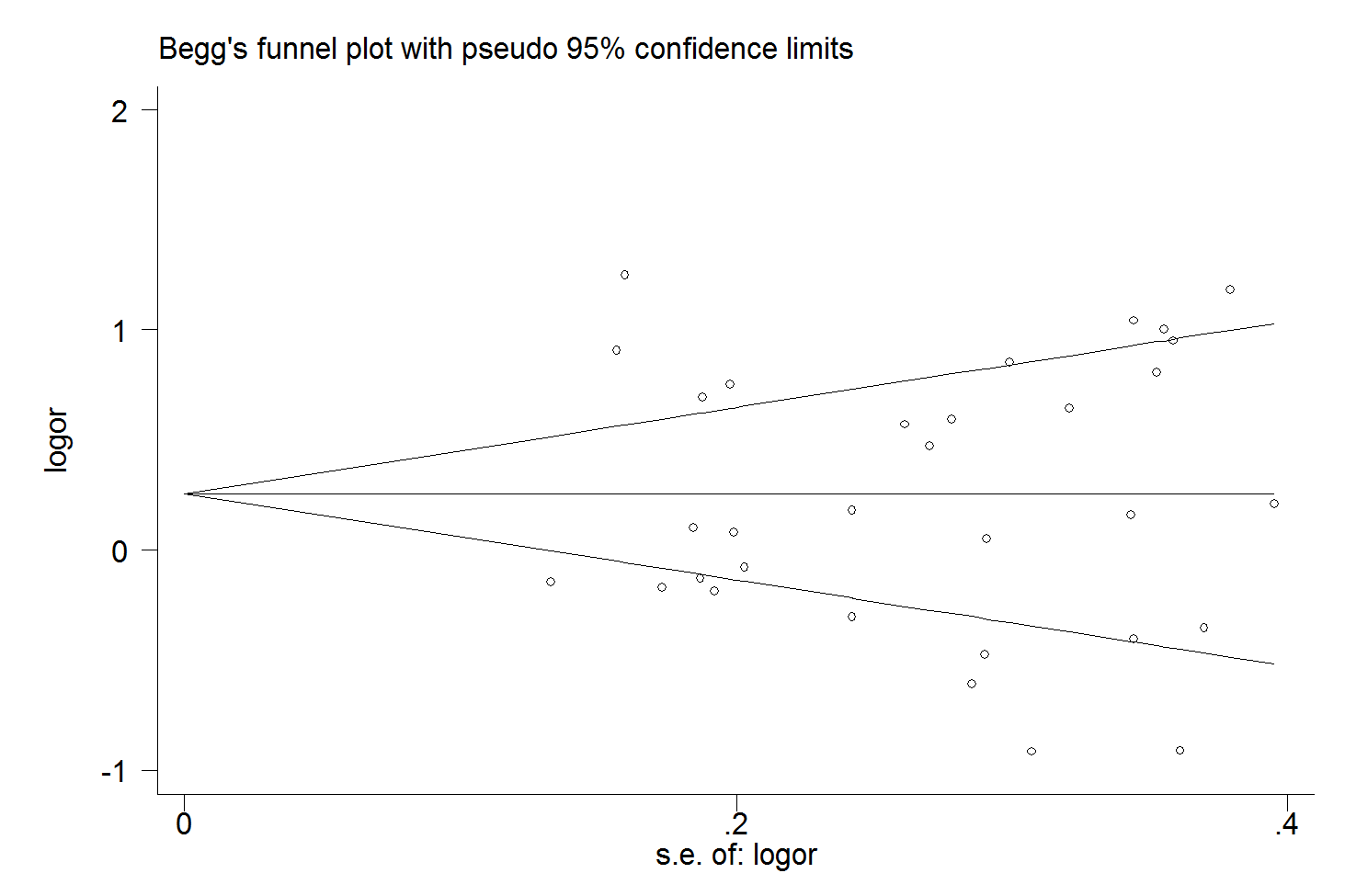

Supplement: Figure S2 — Begg’s funnel plot for publication bias in selection of studies on GSTM1 polymorphism. (TIF) [file pone.0048924.s002.tif]

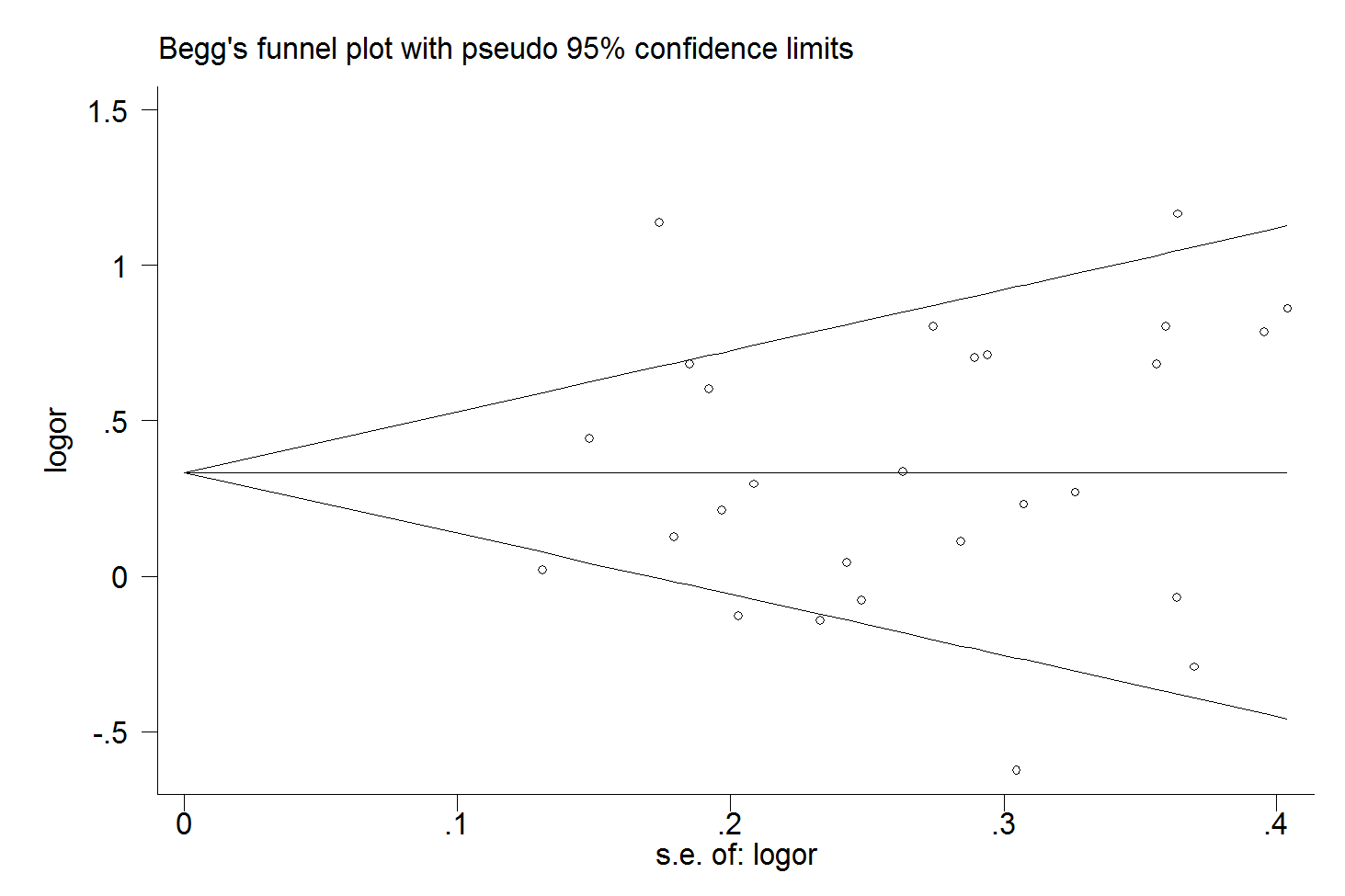

Supplement: Figure S3 — Begg’s funnel plot for publication bias in selection of studies on GSTT1 polymorphism. (TIF) [file pone.0048924.s003.tif]
